# Embracing Artificial Intelligence: Revolutionizing Nursing Documentation for a Better Future

**DOI:** 10.7759/cureus.57725

**Published:** 2024-04-06

**Authors:** Sankalp Yadav

**Affiliations:** 1 Medicine, Shri Madan Lal Khurana Chest Clinic, New Delhi, IND

**Keywords:** healthcare, ai and machine learning, hipaa, nursing documentation, nursing

## Abstract

Nursing documentation stands as a critical aspect of healthcare delivery, ensuring comprehensive patient records and facilitating communication among healthcare providers. However, traditional documentation methods are often time-consuming and prone to errors, diverting nurses' attention from direct patient care. This editorial explores the transformative potential of artificial intelligence (AI) in revolutionizing nursing documentation processes. By leveraging AI-driven technologies, such as natural language processing and machine learning, healthcare organizations can automate data entry, extract key clinical information, and generate personalized care plans, thereby streamlining workflows and improving documentation accuracy. This editorial also examines various AI-powered software applications and platforms that facilitate nursing documentation, highlighting their benefits in terms of efficiency, accuracy, and clinical decision support. Furthermore, it discusses considerations such as privacy, security, and the need for nurse training to effectively integrate AI into nursing practice. By embracing AI in nursing documentation, healthcare organizations can empower nurses to devote more time to patient care while enhancing the quality and safety of healthcare delivery.

## Editorial

In the vast field of healthcare, nursing stands as the cornerstone of patient care. Nurses, with their tireless dedication and compassion, are the guardians of health, advocating for patients and ensuring their well-being. Yet, amidst their noble duties, nurses often find themselves burdened with the tedious task of documentation, a necessary but time-consuming aspect of their profession [1]. However, the dawn of artificial intelligence (AI) offers a promising solution to this longstanding challenge, heralding a new era in nursing documentation and patient care.

Traditionally, nurses spend a significant portion of their time meticulously documenting patient information, ranging from vital signs to treatment plans and medical histories. This process, though crucial for maintaining accurate records and facilitating communication among healthcare providers, often diverts nurses' attention away from direct patient care. Moreover, the manual nature of documentation leaves room for errors, leading to potential lapses in patient safety and quality of care [2].

Here's where AI, a technological marvel that holds the potential to revolutionize nursing documentation, enters. By leveraging machine learning algorithms and natural language processing techniques, AI systems can automate the process of data entry and analysis, thereby streamlining documentation workflows and allowing nurses to focus more on patient interaction and critical decision-making [3]. AI-powered tools can extract relevant information from various sources, such as electronic health records (EHRs), diagnostic reports, and nursing notes, and intelligently organize them into comprehensive patient profiles.

Several software applications utilize AI in nursing documentation, helping streamline workflows, enhance accuracy, and improve patient care. Table 1 lists a few examples.

**Table 1:** Software applications that utilize AI in nursing documentation AI: Artificial intelligence; EHR: Electronic health record Epic Systems Corporation: Verona, Wisconsin, US; Cerner Corporation: Kansas City, Missouri, US; DeepCura AI: San Francisco, California, US; Trusted Health: San Francisco, California, US; Suki AI: Redwood City, California, US; Freed: Santa Rosa, California, US; DeepScribe: San Francisco, California, US; Clinithink: London, England, UK; IBM Watson Health: New York, US; Nuance Communications, Inc.: Burlington, Massachusetts, US; Lindy: San Francisco, California, US

Software applications utilizing AI in nursing documentation	
Epic Systems Corporation's AI-powered documentation tools	Epic is a leading provider of EHR systems used by many healthcare organizations worldwide. Their software incorporates AI-driven documentation tools that assist nurses in capturing and organizing patient information efficiently. These tools utilize natural language processing algorithms to extract key clinical data from nursing assessments, progress notes, and other sources, reducing the time spent on manual data entry and ensuring comprehensive documentation.
Cerner Corporation's CareAware VitalsLink	Cerner offers a range of healthcare technology solutions, including CareAware VitalsLink, which leverages AI to automate the documentation of vital signs. This software integrates with medical devices such as bedside monitors and smart infusion pumps, capturing real-time patient data and transferring it directly into the EHR system. By eliminating manual entry errors and delays, CareAware VitalsLink enhances the accuracy of vital sign documentation and enables nurses to focus more on patient care.
DeepCura	It employs AI-driven algorithms to assist nurses in clinical decision-making and care planning, improving overall patient outcomes through personalized interventions.
Nightingale Notes by Trusted Health	Nightingale Notes is a cloud-based nursing documentation platform designed to streamline clinical workflows and improve documentation accuracy. The software utilizes AI algorithms to analyze clinical data and generate personalized care plans based on evidence-based guidelines and best practices. Nurses can easily document patient assessments, interventions, and outcomes using intuitive interfaces and templates, reducing documentation time and enhancing care coordination.
Suki AI Assistant	Suki is an AI-powered digital assistant specifically designed for healthcare professionals, including nurses. The software utilizes voice recognition technology and natural language understanding to transcribe clinical conversations and documentation in real time. Nurses can dictate patient notes, orders, and instructions using their voice, allowing for hands-free documentation and improved efficiency. Suki's AI algorithms continuously learn from user interactions, providing personalized recommendations and automating repetitive tasks to streamline nursing workflows.
Freed	It integrates AI to analyze clinical data and generate comprehensive patient records, ensuring accuracy and efficiency in documentation.
DeepScribe	It utilizes natural language processing AI algorithms to transcribe and organize clinical notes accurately, saving time for healthcare professionals.
Clinithink's CLiX ENRICH	CLiX ENRICH is an AI-powered clinical natural language processing platform that helps healthcare organizations extract structured data from unstructured clinical notes and documents. The software can analyze nursing narratives, physician notes, and other text-based documentation to identify relevant clinical concepts and populate EHR fields automatically. By standardizing and codifying nursing documentation, CLiX ENRICH improves data accuracy, interoperability, and decision support capabilities within healthcare systems.
IBM Watson Health	It quickly turns clinical conversations into text by using sophisticated algorithms. Note completion is streamlined by this software's seamless integration with EHR systems.
Dragon Medical	Created by Nuance Communications, it ensures compliance with Health Insurance Portability and Accountability regulations by streamlining paperwork chores and offering real-time suggestions.
Lindy	It employs AI to automate documentation tasks, enabling nurses to focus more on patient care by reducing administrative burdens.

These examples illustrate how AI technologies are being integrated into nursing documentation software to enhance efficiency, accuracy, and clinical decision-making. As the field of healthcare continues to evolve, we can expect to see further advancements in AI-driven tools that empower nurses and improve patient outcomes.

One of the most significant advantages of AI in nursing documentation is its ability to enhance accuracy and efficiency. Unlike humans, AI systems can process vast amounts of data swiftly and with minimal errors, reducing the likelihood of transcription mistakes and ensuring the integrity of patient records. Furthermore, AI algorithms can learn from historical data patterns, enabling them to provide valuable insights and predictive analytics that aid in clinical decision support and care planning [4].

Moreover, AI-driven documentation solutions have the potential to improve interdisciplinary communication and collaboration within healthcare teams. By centralizing patient information in a standardized format, these systems facilitate seamless information exchange among nurses, physicians, pharmacists, and other stakeholders, fostering a more integrated approach to patient care. Additionally, AI can assist in real-time monitoring of patient status, alerting healthcare providers to critical changes, and enabling timely interventions.

However, the integration of AI into nursing documentation is not without challenges and considerations. Integrating AI into nursing documentation presents several challenges, particularly concerning ethical and legal considerations. While AI offers the potential to streamline documentation processes and enhance patient care, there are significant concerns regarding privacy, liability, and the potential for bias [3].

One of the primary ethical challenges revolves around patient privacy and data security. AI systems require access to vast amounts of patient data to function effectively. Ensuring that patient information is adequately protected against unauthorized access or misuse is critical. Nurses must navigate complex regulations such as the Health Insurance Portability and Accountability Act in the United States and the General Data Protection Regulation in the European Union to safeguard patient confidentiality while utilizing AI for documentation [4,5].

Moreover, the use of AI in nursing documentation raises concerns about liability and accountability. If AI algorithms make errors or provide incorrect recommendations, who bears responsibility? Nurses may face dilemmas regarding whether to trust AI-generated documentation fully or to rely on their own judgment, potentially leading to legal disputes if adverse events occur [3].

Additionally, there is a risk of bias in AI algorithms, which could perpetuate disparities in healthcare delivery. If AI systems are trained on biased data or algorithms, they may inadvertently discriminate against certain patient populations, leading to unequal treatment. Nurses must critically evaluate the accuracy and fairness of AI-generated documentation to mitigate these biases and ensure equitable care for all patients [3].

Furthermore, the ethical implications of AI extend to issues of autonomy and human oversight. As AI becomes increasingly integrated into nursing workflows, there is a concern that it may diminish nurses' autonomy and decision-making capabilities. Nurses must strike a balance between leveraging AI to improve efficiency while retaining their professional autonomy and clinical judgment [3].

Table 2 outlines the issues associated with integrating AI into nursing documentation, along with suggested solutions.

**Table 2:** Issues associated with integrating AI into nursing documentation, along with suggested solutions AI: Artificial intelligence

Issues	Solutions									
Patient Privacy and Data Security	- Implement robust data encryption protocols.									
	- Limit access to patient data to authorized personnel only.									
	- Ensure compliance with privacy regulations such as HIPAA. - Regularly audit AI systems for data breaches.									
Liability and Accountability	- Establish clear protocols for identifying responsibility in case of AI errors.									
	- Develop mechanisms for nurses to override AI recommendations when necessary.									
Bias in AI Algorithms	- Conduct regular audits to detect and mitigate bias in AI algorithms.									
	- Diversify training data to ensure representation of all patient populations.									
	- Implement transparency measures to disclose the sources of bias in AI systems.									
Diminished Autonomy and Human Oversight	- Provide comprehensive training on AI use and encourage critical thinking skills among nurses.									
	- Ensure that AI systems serve as aids to decision-making rather than replacements for clinical judgment.									
	- Foster a culture that values nurses' expertise and input in AI development and implementation.									

As we discuss AI-driven nursing documentation in healthcare, it is essential to recognize that technology is not a panacea but a powerful tool that complements the skills and expertise of healthcare professionals. AI should augment, rather than replace, the human touch in nursing, preserving the essence of compassionate care that lies at the heart of the profession. By embracing AI in nursing documentation, we have the opportunity to redefine the role of nurses, empowering them to devote more time to what truly matters-delivering exceptional care to patients [5].

In conclusion, integrating AI into nursing documentation offers numerous benefits but also presents significant ethical and legal challenges. Nurses must navigate these complexities to ensure that AI is implemented responsibly, prioritizing patient privacy, fairness, accountability, and maintaining professional autonomy. Collaborative efforts between nurses, healthcare institutions, policymakers, and AI developers are essential to address these challenges effectively and maximize the potential of AI in nursing documentation while upholding ethical standards and legal obligations [3].
